# Prolonged forearm ischemia attenuates endothelium-dependent vasodilatation and plasma nitric oxide metabolites in overweight middle-aged men

**DOI:** 10.1007/s00421-018-3886-z

**Published:** 2018-05-21

**Authors:** Zainie Aboo Bakkar, Jonathan Fulford, Phillip E. Gates, Sarah R. Jackman, Andrew M. Jones, Bert Bond, Joanna L. Bowtell

**Affiliations:** 1 Sport and Health Sciences, College of Life and Environmental Sciences, University of Exeter, Exeter, EX1 2LU UK; 2 University of Exeter Medical School, Exeter, UK

**Keywords:** Forearm occlusion, Endothelial function, Nitrite/nitrate, Peroxiredoxin-2

## Abstract

**Purpose:**

Repeated cycles of endothelial ischemia–reperfusion injury and the resulting respiratory burst contribute to the irreversible pathophysiology of vascular diseases, and yet, the effects of ischemia reperfusion on vascular function, oxidative stress, and nitric oxide (NO) bioavailability have not been assessed simultaneously. Therefore, this study sought to examine the effects of prolonged forearm occlusion and subsequent reperfusion on NO-dependent brachial artery endothelial function.

**Methods:**

Flow-mediated dilatation was measured at baseline and 15, 30, and 45 min after 20-min forearm occlusion in 14 healthy, but physically inactive middle-aged men (53.7 ± 1.2 years, BMI: 28.1 ± 0.1 kg m^−2^). Venous blood samples collected from the occluded arm were analyzed for NO metabolites and markers of oxidative stress.

**Results:**

FMD was significantly depressed after the prolonged occlusion compared to baseline, with a significant reduction 15-min post-occlusion (6.6 ± 0.7 to 2.9 ± 0.4%, *p* < 0.001); FMD remained depressed after 30 min (4.1 ± 0.6%, *p* = 0.001), but was not significantly different to baseline after 45-min recovery (5.4 ± 0.7%, *p* = 0.079). Plasma nitrate (main time effect: *p* = 0.015) and nitrite (main time effect: *p* = 0.034) concentrations were significantly reduced after prolonged occlusion. Plasma catalase activity was significantly elevated at 4- (*p* = 0.016) and 45-min (*p* = 0.001) post-occlusion, but plasma peroxiredoxin 2 and protein carbonyl content did not change.

**Conclusions:**

Prolonged forearm occlusion resulted in acute impairment of endothelium-dependent vasodilatation of the brachial artery for at least 30 min after reperfusion. We demonstrate that this vascular dysfunction is associated with oxidative stress and reduced NO bioavailability following reperfusion.

## Introduction

Repeated cycles of occlusion in the vascular bed followed by reestablishment of perfusion (ischemia–reperfusion injury, IRI) are thought to contribute to endothelial dysfunction via increased generation of superoxide (Bertuglia and Giusti 2003; Eltzschig and Eckle 2011) derived from mitochondrial (Ježek and Hlavatá 2005) and enzymatic sources, such as nicotinamide adenine dinucleotide phosphate (NADPH) oxidase and xanthine oxidase (Ray and Shah 2005; Loukogeorgakis et al. 2010). This is thought to be due to decreased nitric oxide (NO) production or bioavailability (Xia et al. 1996), caused by disruption in endothelial nitric oxide synthase (eNOS) function or excess generation of reactive oxygen species (ROS) and conversion to the highly cytotoxic peroxynitrite (Xia et al. 1996) via reaction of NO with superoxide (Li and Shah 2004).

Several human experimental studies have found that prolonged occlusion reduced forearm blood flow (Pernow et al. 2003; Mayahi et al. 2007; Pleiner et al. 2008) as well as endothelium-dependent vasodilatation assessed via flow-mediated dilatation (FMD) after 15-min reperfusion. This may be due to eNOS uncoupling (Pleiner et al. 2008; De Pascali et al. 2014) and related to increased generation of ROS, as indicated by increased plasma F2 isoprostanes (Davies et al. 2009). However, to date, no studies have measured changes in endothelium-dependent vasodilatation in parallel with NO metabolites and biomarkers of oxidative stress after ischemia–reperfusion. Devan et al. (2011) measured plasma nitrite [NO_2_^−^] and nitrate [NO_3_^−^] concentrations prior to occlusion, but this was not related to the subsequent ischemia–reperfusion-induced attenuation of FMD. Mayahi et al. (2007) measured plasma [NO_2_^−^] and [NO_3_^−^] concentrations following 20-min forearm occlusion using the less sensitive spectrophotometric method (Griess reagent) rather than the more sensitive chemiluminescence assay and found no change from baseline after 15 min of reperfusion, but vascular function was not assessed.

Empirical data on the effects of ischemia–reperfusion (IR) on endogenous antioxidant concentration or activity are scarce. Inhibition of ROS production and/or ROS scavenging via endogenous antioxidants may prevent the eNOS uncoupling that contributes to endothelial dysfunction induced by IR (Bertuglia and Giusti 2003; Pleiner et al. 2008). These scavenging mechanisms may involve superoxide and hydrogen peroxide (H_2_O_2_) metabolising enzymes such as superoxide dismutase (SOD), catalase (CAT), glutathione peroxidase (GPx), and peroxiredoxin (Prx). In isolated perfused mouse hearts subjected to 40 min of ischemia followed by 60 min of reperfusion, Prx2 levels decreased to about 65% of pre-ischemic values implicating oxidative stress, although there was no change in the expression of CAT (Zhao et al. 2009).

Ischemia–reperfusion injuries are relatively common vascular events during general surgical procedures, and present a significant problem for organ transplant (Serracino-Inglott et al. 2001). For instance, myocardial injury was experienced during non-cardiac surgery by 8% of individuals aged over 45 years, with the majority of these events clinically silent (Abbott et al. 2016). Despite this incidence level, and the significant tissue damage resulting from IR leading to irreversible pathophysiology such as myocardial infarction and stroke, our understanding of the aftermath of IR and the availability/efficacy of therapeutic strategies is still poor. For the first time, we have made comprehensive measurements of changes in endothelial-dependent vasodilatation after prolonged forearm occlusion, in tandem with the changes in plasma [NO_2_^−^] and [NO_3_^−^], CAT activity, and Prx2 concentration. We hypothesized that (1) flow-mediated dilatation would be attenuated in tandem with reduced plasma nitrite concentration due to reduced NO bioavailability and (2) oxidative damage would occur after prolonged occlusion as evidenced by elevated plasma CAT activity and protein carbonyl (PC) content.

## Methods

### Participants

Fourteen physically inactive male participants (age 53.7 ± 1.2 years, BMI 28.1 ± 0.1 kg m^−2^, Table 1) were recruited from local residents in Devon, United Kingdom. All participants were non-smokers and ostensibly healthy, with no known history of diabetes, cardiovascular diseases, or musculoskeletal conditions, and were not taking any drugs or nutritional supplements as assessed by questionnaires. The experimental protocols were approved by the University of Exeter Research Ethics Committee prior to the commencement of data collection, and conformed with the Declaration of Helsinki. All participants provided their written informed consent at an initial visit, after which participant height, weight, and blood pressure were measured.

**Table 1:** Participant characteristics

Age, year	53 ± 1
Height, cm	179 ± 1
Weight, kg	90.6 ± 3.5
Body mass index, kg/m^2^	28.1 ± 0.1
Heart rate, beat/min	65 ± 1
Systolic blood pressure, mmHg	139 ± 5
Diastolic blood pressure, mmHg	86 ± 3

Values are expressed as mean ± SE

### Experimental protocol

**Fig. 1:**
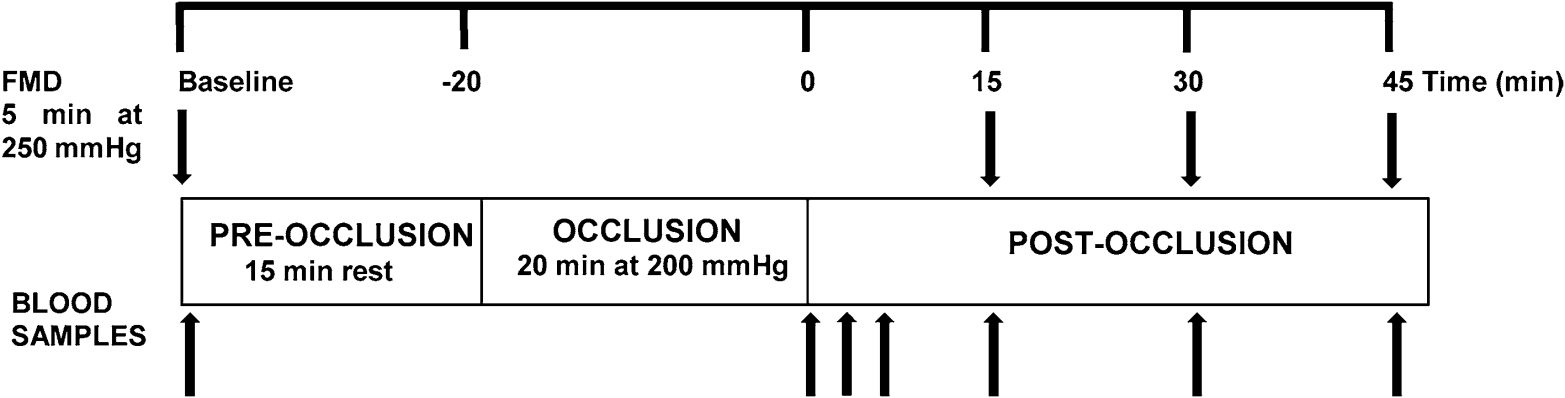
Brachial artery flow-mediated dilatation (FMD) and venous blood collection before and after prolonged occlusion protocol to induce transient ischemic reperfusion injury (IRI). Arrows represent FMD measurements and collection of blood samples

Trials were conducted in a quiet room with dim light and room temperature set at 23–24 °C. A schematic of the experimental protocol is provided in Fig. 1. After an overnight fast and abstention from alcohol, exercise, and caffeine for 48 h, participants arrived at the laboratory and a cannula was placed in the antecubital vein of the left arm. Participants rested in a supine position for 10 min, before basal FMD was assessed in the left arm. After 15-min recovery, the forearm was occluded with a cuff placed just below the elbow and inflated to 200 mmHg for 20 min. The prolonged occlusion procedure was well tolerated by participants. Tissue oxygenation in the forearm musculature was monitored using near infrared spectroscopy (Hamamatsu Photonics KK, Shizuoka Pref., Japan) and tissue oxygenation index (TOI) decreased from 65.1 ± 1.0 to 29.5 ± 2.7% during prolonged occlusion. Most participants reported “pins and needles” in the forearm during reperfusion, but described no other discomfort. FMD measurements were then repeated after 15, 30, and 45-min recovery. Blood samples were obtained at baseline, immediately, and 2, 4, 15, 30, and 45 min after the 20-min occlusion for measurement of plasma [NO_2_^−^] and [NO_3_^−^], with those at 15, 30, and 45 min collected immediately after the cuff release associated with the FMD measurement. Blood samples were centrifuged at 4000 rpm and 4 °C for 10 min, within 1 min of collection. Plasma samples were immediately frozen at − 80 °C for later analysis. Plasma [NO_2_^−^] and [NO_3_^−^] was measured in all samples and plasma CAT activity, protein carbonyl and Prx2 content were measured at baseline and 4- and 15-min reperfusion.

## Measurements

### Brachial artery FMD

Brachial artery dilation in response to a 5-min ischemic stimulus was performed to quantify endothelium-dependent vasodilatation. Brachial artery FMD was examined in the left arm, with the arm extended and positioned at an angle of approximately 80° from the torso. To allow capture of ECG gated images, three ECG electrodes were positioned at the left and right intraclavicular fossa and left iliac crest. A rapid inflation and deflation pneumatic cuff (Hokanson, Bellevue, WA) was positioned around the forearm immediately distal to the olecranon process. A high-resolution ultrasonography system (Sequoia 512, Acuson; Siemens) with a 13-MHz linear array transducer was used to image the brachial artery in the distal one-third of the upper arm in accordance with the established protocol in our laboratory (Bond et al. 2015a, b) and recent guidelines (Thijssen et al. 2011). To ensure arm stability and transducer placement, a customized armrest and transducer holder device cradled the arm and locked the transducer in position providing the optimal image of the brachial artery. Ultrasound parameters were set to optimize the longitudinal B-mode images of the lumen–arterial wall interface and maintained for all recordings. Continuous Doppler velocity measurements were collected using a 60° insonation angle for all acquisitions. The occlusion cuff was inflated to 250 mm Hg to completely block the arterial inflow for 5 min. Brachial artery diameter and blood flow recordings resumed 30 s before cuff deflation and continued for 3 min thereafter. Ultrasound images were captured at the beginning of the R-wave, which marks the end of the diastolic phase and then analyzed off-line using validated electrocardiogram (ECG)-gating software (Brachial Analyzer 5 Vascular Devices, Medical Imaging Applications, Coralville, IA) (Mancini et al. 2002). As an indication of autonomic function, heart rate variability (HRV) was calculated using the root mean square of the squared differences (RMSSD) between adjacent R-R intervals during the 60 s prior to cuff inflation (Kubios, Biosignal Analysis and Medical Imaging Group, Joensuu, Finland), in line with our earlier work (Bond et al. 2016).

### Plasma nitrite and nitrate concentration

All glassware, utensils, and surfaces were rinsed with deionized water to remove residual [NO_2_^−^] and [NO_3_^−^] before blood analyses. Undiluted (nondeproteinized) and deproteinized plasma was used to analyze [NO_2_^−^] and [NO_3_^−^], respectively, using a Sievers gas-phase chemiluminescence NO analyzer (NOA; Sievers NOA 280i; Analytix, Durham, UK) as described in the previous studies (Wylie et al. 2013a, b).

### Plasma protein carbonyl concentration

Protein content in all plasma samples was measured using NanoDrop Lite (Thermo Fisher Scientific Inc, Delaware, USA). Each plasma sample was diluted to 10 µg/ml of protein in 1X phosphate buffer saline, and analyzed for protein carbonyl content (OxiSelect Protein Carbonyl ELISA kit, Cell Biolabs Inc, California, USA). Briefly, samples were allowed to adsorb to wells of a 96-well plate and then reacted with dinitrophenol hydrazine (DNPH). The protein carbonyls derivatized to dinitrophenyl hydrazone (DNP) were probed with an anti-DNP antibody. The standard curve was prepared from commercially prepared reduced and oxidised BSA standards as provided.

### Plasma Prx2 concentration

Plasma was diluted 16-fold and analyzed for [Prx2] using enzyme-linked immunosorbent assay (ELISA) kit according to the manufacturer’s instructions (Cloud-Clone Corp, Texas, USA). Briefly, standards and samples were added to the microtiter plate wells with a biotin-conjugated antibody specific to Prx2. Next, avidin conjugated to horseradish peroxidase was added to each microplate well and incubated. After tetramethylbenzidine substrate solution was added, wells that contain Prx2, biotin-conjugated antibody and enzyme-conjugated avidin exhibited a change in colour. The enzyme–substrate reaction was terminated by the addition of sulphuric acid solution and the colour change was measured spectrophotometrically at a wavelength of 450 ± 10 nm. The concentration of Prx2 in the samples was determined by comparing the optical density of the samples to the standard curve.

### Plasma CAT activity

Plasma was diluted fivefold and analyzed for CAT activity using enzyme-linked immunosorbent assay (ELISA) kit according to the manufacturer’s instructions (Cayman Chemical, Michigan, USA). In brief, the peroxidase function of CAT was determined based on the reaction of the enzyme with methanol in the presence of an optimal concentration of hydrogen peroxide. The change in colour of formaldehyde formation was measured with 4-amino-3-hydrazino-5-mercapto-1,2,4-triazole as the chromogen. One unit of CAT was defined as the amount of enzyme that causes the formation of 1.0 nmol of formaldehyde per minute at 25 °C. CAT activity was expressed in nmol/min/ml.

### Data and statistical analysis

All FMD analyses were performed by the same investigator. The region of interest was carefully defined on the clearest images and this process was repeated for every baseline, cuff inflation, and deflation during FMD measurement. Frame-by-frame analysis of artery diameter (mm) and blood flow velocity (m/s) was performed by the same analyst. Pre diameter was calculated as mean artery diameter during 1 min recording before each cuff inflation. Peak diameter was determined as the highest artery diameter after each cuff deflation. Endothelium-dependent vasodilatation was calculated as the percentage increase in arterial diameter after a 5-min ischemic stimulus. The total shear rate area under the curve (SR AUC) was calculated as the area under the shear curve vs time immediately after cuff deflation until peak arterial diameter (Pyke and Tschakovsky 2006). Baseline FMD was correlated with SR AUC (*r* = 0.712, *p* = 0.004), and therefore, FMD was also normalized to SR AUC (31). All data are presented as mean ± standard error (SE). FMD, baseline diameter, peak diameter, SR AUC, RMSSD as well as plasma [NO_2_^−^] and [NO_3_^−^], [PC], [Prx2], and CAT activity were analyzed using one-way repeated measures analysis of variance (ANOVA) with time treated as a within subject variable. When a significant effect was found, Bonferroni corrected *t* tests were used to determine specific differences. Data were analyzed using statistical software (SPSS Version 23; IBM Corporation, New York, USA), with significance accepted as *p* ≤ 0.05.

## Results

### FMD

**Fig. 2:**
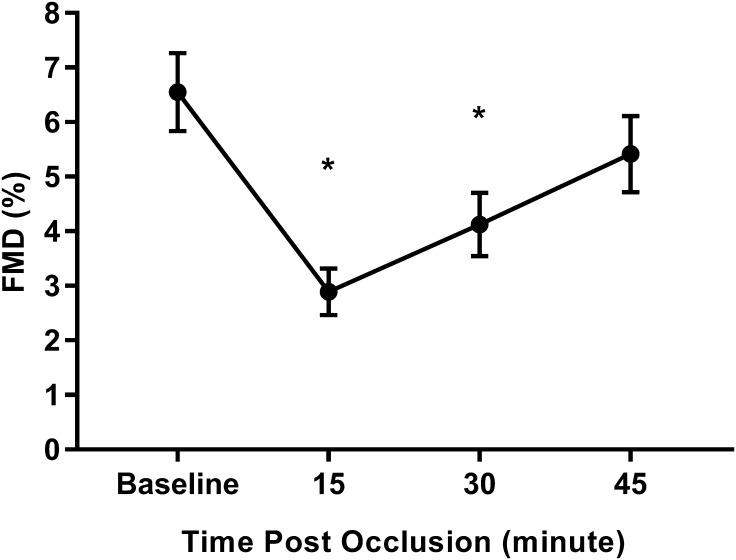
Brachial artery flow-mediated dilatation (FMD) measured before and after prolonged occlusion. *p < 0.05 vs baseline

Flow-mediated dilatation was significantly depressed after the prolonged occlusion compared to baseline (main effect of time, *p* = 0.001; Fig. 2), with FMD significantly lower than baseline after 15 min (56 ± 5%, *p* < 0.001) and 30 min (36 ± 6%, *p* = 0.001) but not 45 min after prolonged occlusion (15 ± 8%, *p* = 0.079). Brachial artery diameter measured before cuff inflation for FMD assessment was significantly higher 15 min after the prolonged occlusion (*p* = 0.005) when compared to baseline values but not at 30- or 45-min post-occlusion (*p* > 0.05, Table 2). In contrast, the peak diameter was significantly lower 15 min (p = 0.009) and 30 min (p = 0.020) after prolonged occlusion when compared to baseline values. There was also a tendency for reduction in SR AUC (main time effect, *p* = 0.095, *η*^2^ = 0.149) after prolonged occlusion compared to baseline (Table 2). FMD normalized to SR AUC (FMD:SR AUC) was also significantly depressed after the prolonged occlusion compared to baseline (main effect of time, *p* < 0.001; Table 2), with FMD:SR AUC significantly lower than baseline after 15- (50 ± 6%, *p* < 0.001) and 30-min (33 ± 5%, *p* = 0.02) post-occlusion but not 45 min after prolonged occlusion (16 ± 7%, *p* = 0.481).

**Table 2:** Brachial artery flow-mediated dilatation measures before (pre) and after (15, 30, and 45 min) prolonged occlusion

Time	Pre diameter (mm)	Peak diameter (mm)	SR AUC	FMD:SR AUC (AU)
Baseline	3.80 ± 0.13	4.04 ± 0.13	694 ± 82	0.98 ± 0.09
15-min post	3.88 ± 0.13*	3.99 ± 0.14*	603 ± 55	0.49 ± 0.06*
30-min post	3.83 ± 0.13	3.98 ± 0.14*	622 ± 49	0.65 ± 0.07*
45-min post	3.80 ± 0.13	4.00 ± 0.13	684 ± 61	0.79 ± 0.08

Data are expressed as means ± SE

*FMD* flow-mediated dilatation, *SR AUC* shear rate area under the curve

*p < 0.05 vs baseline

### HRV

There was no main effect of time for RMSSD (baseline 41.6 ± 3.8 ms; post-occlusion 42.3 ± 4.0 ms; 15-min post-occlusion 41.4 ± 3.0 ms; 30-min post-occlusion 40.3 ± 4.3 ms; 45-min post-occlusion 43.7 ± 4.8; *p* = 0.56).

### Plasma analyses

**Fig. 3:**
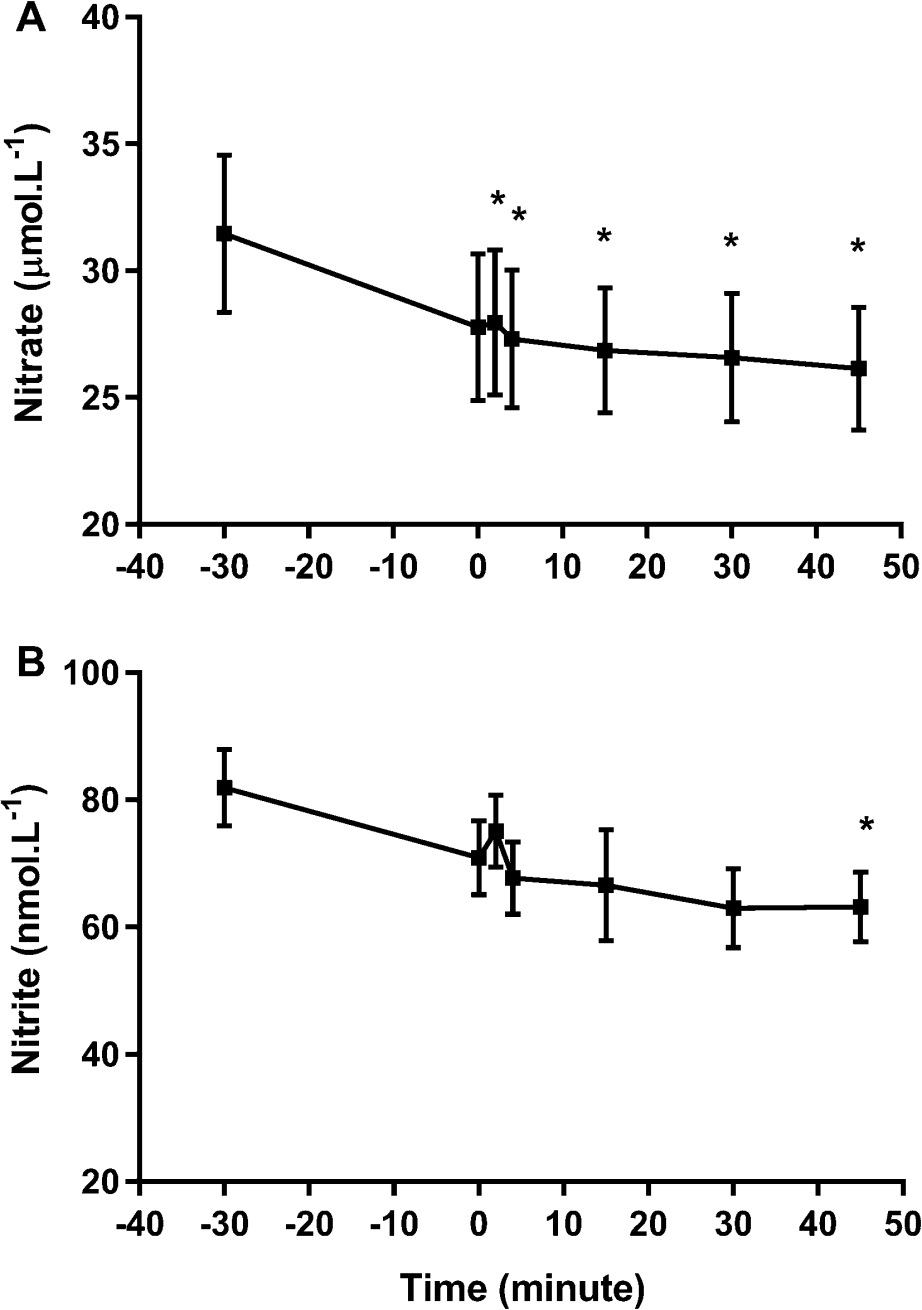
Plasma nitrate (**a**) and nitrite (**b**) concentration measured before and after prolonged occlusion. *p < 0.05 vs baseline

**Fig. 4:**
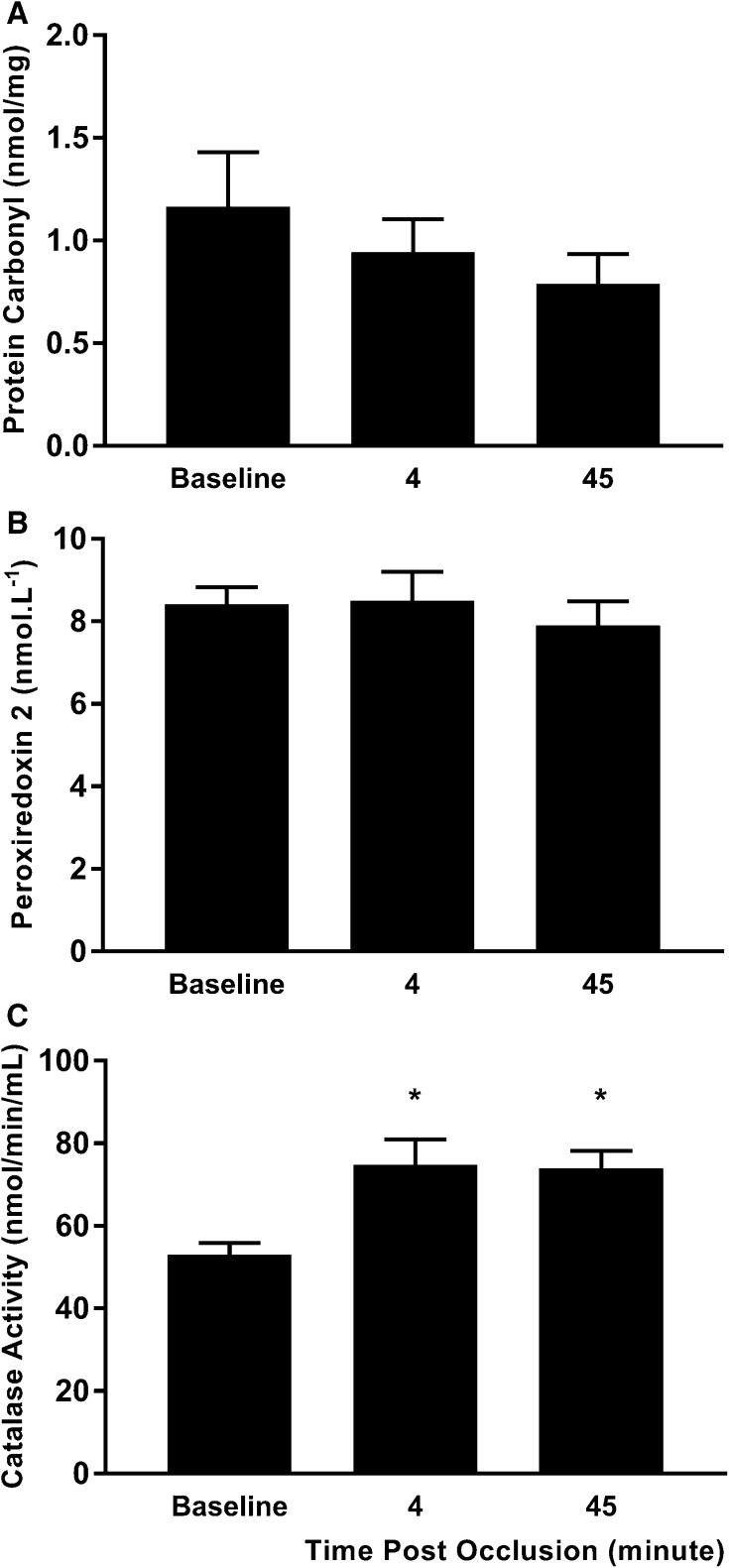
Plasma protein carbonyl concentration ([PC]) (**a**), peroxiredoxin 2 concentration ([Prx2]) (**b**) and catalase (CAT) activity (**c**) measured before and after prolonged occlusion. *p < 0.05 vs baseline

Plasma [NO_3_^−^] (main effect of time, *p* = 0.015, Fig. 3a) and [NO_2_^−^] (main time effect: *p* = 0.034, Fig. 3b) were significantly reduced after prolonged occlusion, with post hoc analysis revealing significant reductions vs baseline at all timepoints for [NO_3_^−^] and at 45 min for [NO_2_^−^]. There were no significant effects of prolonged occlusion on plasma PC (Fig. 4a) and Prx2 (Fig. 4b) contents at any timepoints. However, plasma CAT activity was significantly elevated at 4- (*p* = 0.016) and 15-min (*p* = 0.001) post-occlusion (Fig. 4c).

## Discussion

Endothelium-dependent vasodilatation was impaired for at least 30 min following the prolonged forearm occlusion. Here, we report, for the first time, the effects of experimentally induced IR on HRV, plasma [NO_3_^−^], [NO_2_^−^], PC and Prx2 concentrations as well as CAT activity in parallel with FMD. We demonstrate that the attenuation of FMD occurred in the absence of any changes in autonomic function, but was mirrored by the significant reduction in both plasma [NO_3_^−^] and [NO_2_^−^] concentrations after prolonged occlusion, implicating reduction in NO bioavailability in the impaired vascular response following prolonged IR. This is perhaps due to the oxidative stress induced by the ischemia reperfusion, since plasma CAT activity was significantly elevated post-occlusion, although there were no significant changes in plasma PC and Prx2 concentrations.

In the present study, FMD was reduced by 50% for at least 30 min after prolonged occlusion, but was not statistically different from baseline after 45-min reperfusion. This magnitude of FMD suppression is consistent with data in sedentary adults reported by other groups: 30% (DeVan et al. 2011) and 36% (Rakobowchuk et al. 2013) 15-min post prolonged occlusion, although Rakobowchuk et al. (2013) found that FMD recovered earlier (30-min post-occlusion) in eight healthy middle-aged women. In contrast, Carter et al. (2014) reported a similar duration of suppression, but found that the magnitude of FMD suppression was greater (46%, Carter et al. 2014). This may relate to the cuff placement on the upper arm (Carter et al. 2014), whereas, in the present study and others, the cuff was applied just below the elbow during the 20-min occlusion (DeVan et al. 2011; Rakobowchuk et al. 2013). In the present study, there was a tendency for augmentation in baseline diameter of the brachial artery after prolonged occlusion which was also observed in the previous studies (Carter et al. 2014; Schreuder et al. 2014). Mullen et al. (2001) reported that 15-min hand ischemia resulted in a more prolonged reactive hyperemia and sustained dilation of the artery proximal to the ischemia location in response to a continuous flow stimulus which was unaffected by the NO synthase inhibitor N^G^monomethyl-_L_-arginine (l-NMMA). Therefore, in contrast to the response to a 5-min occlusion, which is largely NO dependent, the vasodilation induced by a more prolonged occlusion appears to also involve NO-independent mechanisms. In support of this view, endothelium-derived hyperpolarizing factor is upregulated during eNOS dysfunction, causing arterial vasodilation via vascular smooth muscle hyperpolarization (Bryan et al. 2005). Previous studies that adopted a similar forearm occlusion protocol also demonstrated that IR causes endothelial but not smooth muscle dysfunction (Pernow et al. 2003; Pleiner et al. 2008). Pernow et al. (2003) showed a significant attenuation in the acetylcholine-induced endothelium-dependent vasodilatation 15 and 30 min after occlusion, but there was no difference in endothelium independent vasodilatation response to sodium nitroprusside (SNP) at 30- and 60-min reperfusion. This suggests that the ability of the smooth muscle to relax was unaffected by IR, although the vascular response to SNP at 15-min post-occlusion was not assessed (Pernow et al. 2003). Pleiner et al. (2008) confirmed that smooth muscle function in response to nitroglycerin was unaffected 15 min after 20-min forearm occlusion, and this pathway may contribute to the increase in prediameter after prolonged occlusion.

Following reperfusion, eNOS activity is reduced resulting in suppressed NO production (Lundberg et al. 2015). Furthermore, the ischemic conditions in the forearm tissue induced by the prolonged occlusion will produce an hypoxic environment (decline from 65 to 29% tissue oxygenation index in the forearm musculature in this study) with low pH in the vasculature distal to the cuff, which will promote reduction of nitrite to NO (Lundberg et al. 2008). The reductions in plasma [NO_2_^−^] and [NO_3_^−^] observed in the present study may, therefore, be due to both decreased nitrite formation from NO produced via eNOS, as well as increased conversion of nitrite to NO. Plasma [NO_2_^−^] and [NO_3_^−^] were reduced after prolonged occlusion and remained depressed after 45-min reperfusion when FMD was no longer statistically different from baseline. This corroborates the suggestion that restoration of FMD after prolonged occlusion is not entirely NO dependent.

Reperfusion following occlusion results in increased ROS generation, with significant increases in plasma F2-isoprostane after prolonged occlusion which may be sustained for up to 4 h (Traustadottir et al. 2009). However, in the present study, there was no change in plasma protein carbonyls or Prx2 concentration after prolonged occlusion. A positive association between plasma Prx2 level and endothelial function has been reported in both human (El Eter et al. 2014) and animal studies (Zhao et al. 2009; Park et al. 2011). Mouse hearts perfused ex vivo in Langendorff model and subjected to 40 min of ischemia followed by 60 min of reperfusion showed a significant decrease in Prx2 to about 65% of pre-ischemic values (Zhao et al. 2009). It is possible that the shorter duration of forearm ischemia (20 min) and reperfusion (45 min), and smaller relative tissue volume involved in the present study as well as higher sensitivity of the myocardium vs the peripheral vasculature to ischemia accounted for the discrepancy in Prx2 response. There was, however, a significant increase in plasma CAT activity after prolonged occlusion in the present study, which is suggestive of increased levels of hydrogen peroxide induced by ischemia–reperfusion. Previous studies with isolated human arterioles suggest that even brief periods of elevated intra-luminal pressure impair NO bioavailability and elevate mitochondrial-derived hydrogen peroxide that provides a compensatory dilator mechanism (Beyer et al. 2014). Interestingly, Akinmoladun et al. (2016) found that SOD activity was significantly reduced, whereas CAT activity was increased in perfusates from reperfused ischemic rat hearts compared to non-ischemic heart perfusates (Akinmoladun et al. 2016). Catalase has a higher affinity for H_2_O_2_ at higher concentrations (*Km* for H_2_O_2_ approximately 1 mM) when compared to Prx2 (*Km* for H_2_O_2_ approximately 1 µM) (Chae et al. 1999; Vetrano et al. 2005), which may account for the differential CAT and Prx2 response to IR in the present study.

In conclusion, prolonged occlusion resulted in a temporary reduction in endothelium-dependent vasodilatation of the brachial artery with response returned to baseline within 45 min. Ischemia induced reductions in vascular function are likely to be related in part to reductions in NO bioavailability due to ROS generation following reperfusion. Therapeutic approaches to combat IR and its consequences should, therefore, include enhancing antioxidant system capability and/or NO bioavailability.

## Abbreviations

CAT: Catalase
DNP: Dinitrophenol hydrazine
ECG: Electrocardiogram
ELISA: Enzyme-linked immunosorbent assay
eNOS: Endothelial nitric oxide synthase
FMD: Flow-mediated dilatation
GPx: Glutathione peroxidase
IRI: Ischaemia reperfusion injury
NO: Nitric oxide
NO_3_^−^: Nitrate
NO2^−^: Nitrite
NADPH: Nicotinamide adenine dinucleotide phosphate
Prx: Peroxiredoxin
PC: Protein carbonyl
ROS: Reactive oxygen species
SNP: Sodium nitroprusside
SOD: Superoxide dismutase
